# Nonimmune Hydrops Fetalis—Prenatal Diagnosis, Genetic Investigation, Outcomes and Literature Review

**DOI:** 10.3390/jcm9061789

**Published:** 2020-06-08

**Authors:** Przemyslaw Kosinski, Pawel Krajewski, Miroslaw Wielgos, Aleksandra Jezela-Stanek

**Affiliations:** 1 1st Department of Obstetrics and Gynecology, Medical University of Warsaw, 02-015 Warsaw, Poland; mwielgos@wum.edu.pl; 2 Neonatal Unit, 1st Department of Obstetrics and Gynecology, Medical University of Warsaw, 02-015 Warsaw, Poland; pkrajewski1@wum.edu.pl; 3 Department of Genetics and Clinical Immunology, National Institute of Tuberculosis and Lung Diseases, 01-138 Warsaw, Poland; jezela@gmail.com

**Keywords:** nonimmune foetal hydrops, genetic syndromes, prenatal evaluation, neonatal outcomes

## Abstract

The aim of this paper is to review the outcomes and discuss the genetic and non-genetic aetiology of nonimmune hydrops fetalis in order to support differential ultrasound and genetic evaluations and family counselling. This single-centre study includes all cases of nonimmune hydrops fetalis diagnosed prenatally from 2009 to 2019. Two sources of data were used for this study (prenatal and neonatal) to compare and summarise the findings. Data from genetic testing and ultrasound scans were collected. In total, 33 pregnant women with prenatally diagnosed nonimmune hydrops fetalis were studied. The data included 30 cases of singleton (91%) and three cases (9%) of twin pregnancies. There were 14 survivors (43%), seven cases of postnatal deaths (21%), four cases of intrauterine foetal demises (12%), four cases of termination of pregnancy (12%), and four women without a follow up (12%). The total number of chromosomally normal singleton pregnancies was 29 (88%), and 14 foetuses had an anatomical abnormality detected on the ultrasound scan. The chance of survival was the highest in cases of isolated, idiopathic hydrops fetalis, which in most cases was due to an undetectable intrauterine infection. In many cases, the diagnosis could not be established throughout pregnancy. Each case of nonimmune hydrops fetalis should thus be analysed individually.

## 1. Introduction

The prevalence of nonimmune hydrops fetalis (NIHF) in the general population is estimated to be 1 in every 2500–3500 neonates and 1 in every 1600–7000 foetuses [1]. Hydrops fetalis is the result of an imbalance in the regulation of fluid leading to an increase in interstitial fluid production or a decrease in lymphatic return. By definition, hydrops fetalis involves an accumulation of fluid in the foetal soft tissues and serous cavities. These features are detected by prenatal ultrasound and are defined as the presence of more than two abnormal fluid collections in the foetus. Nonimmune hydrops fetalis refers specifically to cases not caused by red cell alloimmunization.

Disorders or mechanisms that can lead to NIHF include cardiovascular (21.7%), idiopathic (17.8%), genetic (13.4%), haematological (10.4%), infectious (6.7%), and metabolic (1.1%) issues, as well as chest tumours (6.7%), urogenital issues (2.3%), monochorionic twin pregnancy (and related complications (TTTS, TRAP)) (5.6%) and gastrointestinal problems (0.5%) [1,2]. The prevalence of the potential causes of NIHF depends on gestational age at the time of presentation [3,4]. In most cases of early NIHF, the underlying cause is a chromosomal aneuploidy or a complex developmental anomaly, while cardiovascular, urinary and gastrointestinal malformations are diagnosed more commonly during later gestations (>22 weeks). The low incidence of NIHF has led to a limited number of publications on the outcomes of pregnancies with hydrops fetalis. Therefore, the aim of this study was to determine the neonatal outcomes and prevalence of genetic or structural abnormalities among a group of foetuses with prenatally diagnosed hydrops fetalis.

## 2. Experimental Section

This study uses data based on 10 years of experience with prenatally diagnosed NIHF between 2009 and 2019 at our institution, which is a tertiary referral centre for foetal abnormalities. The vast majority of cases were referred to our centre due to suspected abnormalities or diagnosed hydrops fetalis. All women had genetic tests performed (karyotype or chromosomal microarray analysis (CMA)), which were clinically verified after birth. All women had their medical history taken and blood tests performed for TORCH (toxoplasmosis, rubella, cytomegalovirus and Herpes simplex virus) screening. We also recorded each woman’s maternal age, gestational age at diagnosis and pregnancy outcomes (gestational age at delivery and neonatal outcome). Only cases without serum antibodies were included to confirm the nonimmune aetiology of hydrops. In all cases, ultrasound scans were performed to evaluate foetal growth, anatomical defects, placental abnormalities (particularly oedema) and amniotic fluid volume, while Doppler studies were used to examine the middle cerebral artery peak systolic velocity (MCA PSV), the pulsatility index (MCA PI), the umbilical artery pulsatility index (UmA PI) and the ductus venosus pulsatility index (DV PI). All ultrasound scans were performed by experienced operators using a Voluson E6 ultrasound machine (GE Healthcare, Tiefenbach 15, 4871 Tiefenbach, Austria). Data from the foetal ultrasound findings were recorded and analysed. Data are expressed as the median (range). All statistical analyses were performed with the commercially available computer software PASW Statistics 18 (SPSS Inc., Chicago, IL, USA).

This was a retrospective cohort study of women treated routinely in one medical institution. As a descriptive, retrospective study, institutional ethics committee permission was not necessary, as this paper does not report on any primary research. All the data analysed were collected as part of routine diagnosis and treatment, and the data were collected over the last 10 years.

## 3. Results

**Figure 1:**
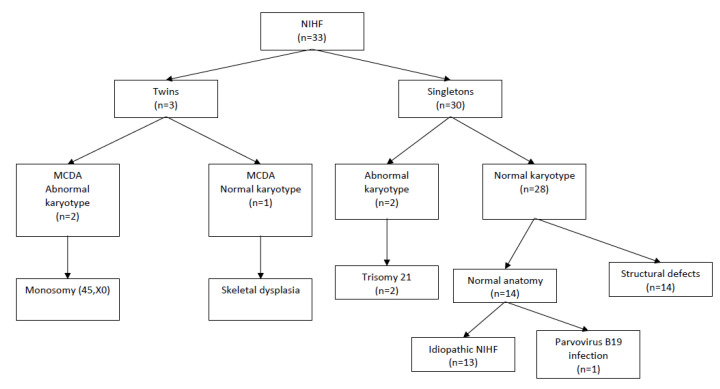
Study population flowchart. NIHF, nonimmune hydrops fetalis; MCDA, monochorionic diamniotic twin pregnancy.

Data from 33 pregnant women with prenatally diagnosed NIHF were collected. These data include 30 cases of singleton (91%) and three cases (9%) of twin pregnancies (all cases featured monochorionic diamniotic twins (MCDA), and only one foetus had hydrops). None of the MCDA twin pregnancies presented symptoms of twin-to-twin transfusion syndrome (TTTS). The median maternal age was 32 (range: 16–46 years). The median gestational age at the time of diagnosis was 24 weeks (range: 15–36 weeks). In all cases, prenatal or (in cases where the gestational age was too advanced for invasive procedures) postnatal genetic testing was performed. In most cases, in accordance with recommended prenatal diagnostic procedures, only classical karyotyping was available. However, all liveborn children were followed up during the neonatal period to verify the need for further genetic testing. In total, four cases (12%) featuring chromosomal aberrations, including two cases of trisomy 21, and two cases of MCDA pregnancies with one foetus with hydrops and abnormal karyotypes (45, X) were confirmed. The total number of chromosomally normal singleton pregnancies was 29 (88%), and 14 foetuses had anatomical abnormalities detected on the ultrasound scan. The study group flow chart is presented in Figure 1. Among the foetuses with detected anatomical defects, the most common types of abnormality were congenital diaphragmatic hernias (28.5%), heart defects (14.2%), and skeletal dysplasia (14.2%). The types of structural anomalies detected in foetuses with NIHF are presented in Table 1.

One case featured a confirmed parvovirus B19 infection treated with an early intrauterine blood transfusion (haemoglobin concentration before transfusion: 2.2 g/dL). There were four in utero foetal demises: one due to severe anaemia from parvovirus B19 infection (foetal demise at 19 weeks), one case of sacrococcygeal teratoma (SCT), a partial hydatidiform mole pregnancy and one case of skeletal dysplasia. Four women decided to terminate their pregnancy (two cases of trisomy 21 with a foetal akinesia deformation sequence and one case of hypoplastic heart syndrome), and four did not follow up. In seven cases, there were postnatal deaths (mostly due to structural defects). There were four cases of congenital diaphragmatic hernia, one case of vein of Galen aneurysmal malformation, one case of polycystic kidney disease and one case of idiopathic hydrops fetalis. All cases of severe hydrothorax included either intrauterine pleural–amniotic shunt placement or a single puncture to reduce pleural effusion (in cases where shunt placement was not possible due to technical limitations). The median gestational age at the time of delivery was 32 weeks (range 29–37 weeks), and the average birthweight was 2495 g (range: 1850–3440 g). In the vast majority of cases, the time of delivery was based on a foetal wellbeing assessment including Doppler studies. All known survivors (43%) presented cases of idiopathic hydrops fetalis with a normal karyotype.

## 4. Discussion

Fewer than half of the isolated cases of nonimmune hydrops fetalis in our cohort resulted in a liveborn baby being discharged home with its mother. This has also been described by other researchers [5] and indicates that the prognosis of NIHF depends on many factors, including the underlying aetiology, the gestational age at detection and delivery, the need for resuscitation in the delivery room, and whether the newborn needs transportation to more specialised medical units [6]. In another prenatal series of 71 pregnancies, the survival rate was approximately 50%, and only 25% survived without major morbidities [7]. In all cases of hydrops fetalis, a thorough ultrasound scan should be examined to exclude foetal abnormalities. It is always a diagnostic challenge to establish the aetiology of NIHF, which is essential for counselling. This may be accomplished only if a systematic approach is applied to exclude possible causes, factors severely influencing the mortality rate, and other elements that are important when medical professionals talk to parents, such as genetic evaluations, ultrasound assessments, prognoses and possible therapies.

### 4.1. Genetic Evaluation

#### 4.1.1. Chromosomal Aberrations

There are two main types of chromosomal abnormalities: numerical and structural aberrations. Numerical aberrations are usually caused by a failure of chromosome divisions and result in the presence of (an) extra chromosome(s) or the absence of (a) chromosome(s). Common types of numerical aberration include triploidy, trisomy and monosomy (also called aneuploidies). Aneuploidies are responsible for 7% to 16% of NIHF cases [6]. The frequency of a particular aberration varies. Monosomy X (Turner syndrome), accounting for 42% to 67% of aneuploid cases, is the most common [8,9]. Other aneuploidies associated with hydrops include trisomy 21—Down syndrome (23% to 30%); trisomy 13, 18 and 12 (10%); and ploidies (triploidies and tetraploidies) [8,10]. In our case series, 50% (2/4) of chromosomal abnormalities were monosomy X, and the remaining 50% (2/4) were trisomy 21. In another review by Sparks et al., among the 44/65 foetuses tested for a karyotype and/or chromosomal microarray, only monosomy X (six cases), trisomy 21 (three cases) and trisomy 18 (one foetus) were confirmed. Moreover, one balanced paracentric inversion of chromosome 2 was identified but was not verified as pathogenic due to a lack of parental karyotypes [9].

Structural aberrations occur due to a loss/gain or rearrangement of genetic material within a particular chromosome. These aberrations include deletions, duplications, inversions, ring formations and translocations. Detecting such abnormalities is possible with modern genomic medicine. A chromosomal microarray analysis (CMA) is one of the technologies developed for the detection of chromosomal imbalances. This assay involves genomic DNA isolation from a test sample and a control sample, the labelling of the two DNA samples with different fluorochromes, and the cohybridisation of the two differentially labelled DNAs with the target DNA, which represents pieces of the human genome.

Depending on the size of the deleted or duplicated fragment, these aberrations may or may not be identified in standard karyotyping. The small ones (<5 Mb, submicroscopic chromosomal imbalances) called copy number variants (CNVs) are detected via a prenatal chromosomal microarray analysis (CMA). The largest multi-centre prospective trial to date showed that among chromosomally normal pregnancies, approximately 6% that used foetal ultrasound anomalies and 1.7% that used routine indications had clinically significant copy number variants [11]. Shortly after this paper, in 2013, the American Congress of Obstetricians and Gynecologists (ACOG) and the Society for Maternal and Fetal Medicine (SMFM) published a joint statement that recommends CMA as a first-line test when a prenatal ultrasound shows one or more major foetal abnormalities [12]. In line with the latest ACOG and SMFM guidelines for prenatal diagnosis, a chromosomal microarray should be offered to all women undergoing prenatal diagnostic testing regardless of the presence of foetal malformations [13]. This microarray facilitates the detection of at least 2.5% of women whose indications did not include a structural foetal abnormality.

However, based on our experience and literature review, classical karyotyping seems to have significant and sufficient diagnostic value for NIHF. Furthermore, as CMA does not detect balanced chromosome rearrangements that may have clinical significance, alterations in the ploidy level (such as triploidy or tetraploidy) may not detect a clinically significant mosaicism, so a concurrent chromosome analysis should still be performed [14].

#### 4.1.2. Monogenic Syndromes

A syndrome is a disorder characterised by a set of associated symptoms. When the aetiology refers to gene mutations, the term “monogenic syndrome” is preferred. According to the largest review, syndromes account for 5% to 10% of NIHF cases [15]. This classification is based on either molecular (gene sequencing) or phenotypic/clinical findings from ultrasounds/physical examinations/autopsies. Recently described monogenic/multiple malformation syndromes with nonimmune hydrops and proven genetic aetiologies are listed in Table 2.

In order to reliably estimate the frequency of monogenic diseases in the aetiology of NIHF, especially in foetuses with developmental defects, it is justified to perform gene panel or even whole exome sequencing. It is very likely that some of the “syndromes” or “malformations” classified in the literature actually have a genetic basis (which has not been identified) and result from CNVs or pathogenic variants in a given gene. Thus, for the classification of NIHF with a proven genetic cause, we propose using etiological nomenclature, such as “chromosomal aberrations” and “monogenic disorders”. With the advancement in molecular prenatal testing, this nomenclature seems the most reasonable.

The misclassification of syndromic vs. non-syndromic disorders may serve inborn errors of metabolism (IEM). Some of these disorders—particularly lysosomal storage disorders (LSDs)—may antenatally manifest only with hydrops fetalis [17]. These disorders are distinguished from monogenic syndromes by the absence of internal organ malformations, which is why they are listed separately in the NIHF classification. LSDs comprise a heterogeneous group of about 40 disorders that account for up to 25% of NIHF cases [6]. In the largest systematic review of the literature evaluating the incidence and types of lysosomal storage disorders for nonimmune hydrops, the overall incidence of LSD was 5.2% in all NIHF cases tested for any LSD, 17.4% in idiopathic NIHF cases, and 24.6% in idiopathic NIHF cases for which a comprehensive LSD workup was done [18]. The three most commonly diagnosed LSDs are mucopolysaccharidosis type VII (MPSVII, caused by a mutation in the gene encoding beta-glucuronidase), Gaucher disease (GD, caused by a mutation in the gene encoding acid beta-glucosidase) and GM1-gangliosidosis (caused by a mutation in the gene encoding beta-galactosidase-1). From a clinical point of view, the antenatal recognition of these/any LSDs is of great importance. The reasons are as follows: Firstly, there is a 25% recurrence risk in each subsequent pregnancy (apart from MPSII, which is an X-like disease); secondly, the early initiation of postnatal treatment may significantly delay the disease’s progression; and thirdly, there is a risk of early death (before the diagnosis is established). Prenatal diagnostics should thus be performed especially in families with cases of NIHF recurrence and when NIHF occurs in a structurally normal foetus. As the recurrence risk in each subsequent pregnancy may be as high as 25%, it is of great importance to make an accurate diagnosis that also allows targeted molecular prenatal testing.

Thus, if the classical karyotyping or CMA produce normal results, in all pregnancies with NIHF, a detailed ultrasound for monogenic diseases should be performed—the latter covering the panel of genes related to foetal hydrops (which differs among laboratories) or even the whole exome (whole exome sequencing testing, WES), which gives a chance to provide novel, unpredictable diagnoses in addition to known causes of nonimmune hydrops. WES, like any other type of genetic testing, requires individual pre-testing and genetic consultations to discuss its disadvantages (such as results of unknown significance) and to obtain informed consent for the study. Other aetiologies include inherited conditions, such as hemoglobinopathies (the most common being alpha thalassemia), as well as acquired conditions, such as haemolysis, feto-maternal haemorrhaging, parvovirus infection, G-6-PD deficiency, erythrocyte enzymopathies—such as pyruvate kinase deficiency, and maternally acquired red cell aplasia [19].

As presented in Table 2, for pregnancies diagnosed with heart anomalies, especially hypertrophic cardiomyopathy or pulmonary valve stenosis, Noonan syndrome and other Rasopathies should be considered (Swearingen et al., 2019) [20].

### 4.2. Ultrasound in NIHF

The cause of hydrops can be determined in about 60%–85% of cases; however, this percentage includes postnatal evaluation. Thus, accurate prenatal diagnoses of the causes of NIHF are less common [20]. Women with diagnosed hydrops fetalis need regular ultrasound follow ups. In cases of hydrothorax or ascites, the volume of fluid should be measured and monitored. The most common causes of NIHF are cardiovascular diseases. Therefore, a cardiac scan should also be offered. The most common mechanism of hydrops in cardiac abnormalities is congestive heart failure or foetal cardiac arrhythmia (including supraventricular tachycardia or congenital heart block) [21,22]. In selected cases of supraventricular tachycardia (SVT), there is the possibility of transplacental treatment, so the overall prognosis is good.

Other possible ultrasound-detectable causes of NIHF include anatomic abnormalities and congenital infections. In cases of a mediastinal shift, a major impairment in the venous return and cardiac output can lead to hydrops fetalis. On the other hand, gastrointestinal obstruction and infarction may lead to decreased colloid osmotic pressure due to protein loss, making the prognosis less favourable. Pregnancy evaluation also includes screening for viral, bacterial and parasitic infectious diseases, including parvovirus, cytomegalovirus, syphilis and toxoplasmosis [23,24]. Parvovirus is the most common infectious cause of NIHF. Parvovirus B19 has a predilection for erythroid progenitor cells, leading to the inhibition of erythropoiesis and subsequent anaemia and hydrops. Ultrasound monitoring includes a peak systolic velocity assessment of the middle cerebral artery (MCA PSV) to predict anaemia. In cases of a PSV above 1.5 MoM, diagnostic foetal blood sampling is recommended.

### 4.3. Foetal Therapy in NIHF

In some rare cases of nonimmune hydrops fetalis, foetal therapy is possible and may improve the outcome. In cases of cardiac tachyarrhythmia, supraventricular tachycardia, atrial flutter or atrial fibrillation, transplacental treatment with antiarrhythmic drugs is possible. Transplacental therapy can also be offered with betamethasone or dexamethasone if the NIHF is a result of a microcystic congenital pulmonary airway malformation. In cases of suspected foetal anaemia, regardless of the cause, foetal blood sampling followed by intrauterine transfusion is recommended. If the NIHF is related to foetal hydrothorax, chylothorax, a large pleural effusion associated with bronchopulmonary sequestration, or a macrocystic congenital pulmonary airway malformation, needle drainage of the effusion or placement of a thoracoamniotic shunt is possible. However, in cases of foetal hydrops detected before 20 weeks of gestation, shunting is usually technically extremely difficult or even impossible. In this study, the median gestational age at the time of diagnosis was 24 weeks (range: 15–36 weeks). In the recent study by Sileo et al., who analysed a group of 279 foetuses with hydrops, the aetiology of NIFH was suggested to vary significantly depending on the gestational age at the time of diagnosis [25]. This was also observed in our group, as the most common causes of NIHF in the earliest gestational group were aneuploidy and heart defects. In these cases, shunting is usually not recommended.

## 5. Conclusions

The short-term and long-term prognosis both depend on the aetiology of NIHF. In cases where treatment is available, the prognosis depends on response to therapy. Aneuploidy is related to a poor prognosis, and even in the absence of aneuploidy, neonatal survival is often less than 50%. Based on the data from our cohort and the available literature, only isolated cases or potentially treatable causes of NIHF, such as foetal arrhythmia or infection with parvovirus B, have a better prognosis. As the number of potential genetic causes of NIHF is significant, genetic evaluation is crucial and should include classical karyotyping or CMA (to exclude chromosomal aberrations, especially when internal organ malformations are noted) and/or gene sequencing to diagnose monogenic syndromes. Based on the literature and our experience, monogenic abnormalities seem to contribute to diseases presenting with NIHF to a greater extent than chromosomal anomalies; therefore, the diagnosis of such abnormalities is essential. This is of great importance, especially in families with recurrent NIHF, where the probability of chromosomal aetiology is low.

## Figures and Tables

**Table 1:** Structural anomalies detected via ultrasound in foetuses presenting with hydrops.

Type of Anomaly	n
**CDH**	4
**CHD**	2
**Skeletal Dysplasia**	2
**VGAM**	1
**SCT**	1
**CCAM (type 1)**	1
**Partial Hydatidiform Mole Pregnancy**	1
**Polycystic Kidney Disease**	1
**FADS**	1

CDH, congenital diaphragmatic hernia; CHD, congenital heart defect; VGAM, vein of Galen aneurysmal malformation; SCT, sacrococcygeal teratoma; CCAM, congenital cystic adenomatoid malformation; FADS, foetal akinesia deformation sequence.

**Table 2:** Several syndromes related to nonimmune hydrops.

Reference	Syndrome/Phenotype (No. of Cases)	Gene/Aetiology (Methods)
Laterre et al., 2018	Noonan syndrome (2)	*PTPN11*
	Miller–Dieker syndrome (1)	del17p13.3 (thus should be rather classified as chromosomal aberration)
	Neu–Laxova syndrome (1)	*PSAT1*
Sparks et al., 2018	Noonan syndrome (2)	*PTPN11* (target sequencing)
	Generalised lymphatic dysplasia (2)	*PIEZO1* (exome sequencing and target testing in DNA from previous pregnancy)
Hartge et al., 2015 [16]	Mucopolysaccharidosis (3, no type mentioned)	No genetic data
